# Integration of a low‐cost electronic nose and a voltammetric electronic tongue for red wines identification

**DOI:** 10.1002/fsn3.1730

**Published:** 2020-06-29

**Authors:** Fangkai Han, Dongjing Zhang, Joshua H. Aheto, Fan Feng, Tengfei Duan

**Affiliations:** ^1^ School of Biological and Food Engineering Suzhou University Anhui China; ^2^ School of Food and Biological Engineering Jiangsu University Zhenjiang China

**Keywords:** colorimetric sensors, electronic nose, electronic tongue, extreme learning machine, red wine

## Abstract

The purpose of this present study was to develop a rapid and effective approach for identification of red wines that differ in geographical origins, brands, and grape varieties, a multi‐sensor fusion technology based on a novel cost‐effective electronic nose (E‐nose) and a voltammetric electronic tongue (E‐tongue) was proposed. The E‐nose sensors was created using porphyrins or metalloporphyrins, pH indicators and Nile red printed on a C2 reverse phase silica gel plate. The voltammetric E‐Tongue with six metallic working electrodes, namely platinum, gold, palladium, tungsten, titanium, and silver was employed to sense the taste of red wines. Principal component analysis (PCA) was utilized for dimensionality reduction and decorrelation of the raw sensors datasets. The fusion models derived from extreme learning machine (ELM) were built with PCA scores of E‐nose and tongue as the inputs. Results showed superior performance (100% recognition rate) using combination of odor and taste sensors than individual artificial systems. The results suggested that fusion of the novel cost‐effective E‐nose created and voltammetric E‐tongue coupled with ELM has a powerful potential in rapid quality evaluation of red wine.

## Graphical abstract

To develop a rapid and effective approach for identification of red wines that differ in geographical origins, brands, and grape varieties, a multi‐sensor fusion technology based on a novel cost‐effective electronic nose (E‐nose) and a voltammetric electronic tongue (E‐tongue) was proposed. The study demonstrates that fusion of the novel cost‐effective E‐nose and voltammetric E‐tongue in conjunction with ELM was able to identify the tested red wines, which has superior directive significance for rapid quality control of red wine.

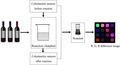

## 1 INTRODUCTION

Red wine is among the best known alcoholic drinks worldwide by virtue of its unique flavor and many vital health benefits, such as anti‐inflammatory and antihypertensive, and reducing the risk of certain diseases including cardiovascular disease and cancer (Sun et al., 2020). Considering that chemical reactions among the key components of wine, that is, water, ethanol, and acids cause the product to change over time (Gil‐Sánchez et al., 2011), there is a pressing need to establish a rapid and effective analytical technique for quality evaluation of red wines to control the winemaking process, avoid counterfeiting, and provide safety assurance for consumers.

Quality evaluation of red wines has always been a complex research area because the quality differences among wines are largely dependent on constituents present in very small proportions (Gil‐Sánchez et al., 2011). Traditional techniques for red wine quality determination are mainly based on chemical analysis and a sensory panel composed of trained experts. Regarding chemical analysis, contents of alcohol, total sugar, dry extract, and volatile acid are the main monitoring indicators for red wine. Conventional analytical techniques of distillation, titration, or high performance liquid chromatography are the most frequently used to analysis the existence or levels of those indicators. Although these conventional techniques provide detailed and accurate information on individual specific components of the wine, they are faced with some inefficiencies. These include their inability to produce holistic quality assessment for red wine, which emerges from the synergistic effects of various chemicals present in the wine complex represented. Moreover, chemical analysis is usually tedious, time consuming, and incapable be employed straightforwardly (Han, Huang, Teye, Gu, & Gu, 2014).

Sensory evaluation has always been the most effective way to acquire the holistic quality information of food products. However, results from sensory evaluation are subjective because human smell and taste assessments are affected by many factors, such as individual variability of the personnel involved, and decrease insensitivity due to prolonged exposure, fatigue, and variable mental states (Banerjee, Tudu, Bandyopadhyay, & Bhattacharyya, 2016). It is also costly to build and maintain a sensory evaluation laboratory and train the sensory experts. For these reasons, volatile perception based on electronic nose (E‐nose) and taste perception based on electronic tongue (E‐tongue) as cost‐effective solution have been introduced as a reliable substitute for human analysis to assess the holistic quality of foods through odor and taste evaluation and also to obtain more objective results.

E‐nose system mainly comprises an array of gas sensors and a pattern prediction unit, which could be employed to quantify and classify foodstuffs containing complex volatile organic compounds (VOCs). E‐nose sensors mimic the mammalian olfactory receptors response to odor released by a target food material with characteristics of weak selectivity, cross sensitivity, and nonspecific determination. The pattern prediction unit performs identification and discrimination to mimic the mammalian brain for signals processing and finally, a fingerprint of food holistic quality based on odors could be obtained.

During the last decades, diverse E‐nose systems have been researched on quality evaluation of red wine. Specifically, Rodriguez Gamboa, (2020) applied E‐nose to detect wine spoilage threshold; Bellincontro, (2013) classified different types of wines from the same variety of grape and viticultural zone; Others include the quantitative analysis of wine aroma volatiles (Capone et al., 2013); monitoring of wine aging processes (Prieto, 2012); classification among different wines of the same variety of grapes come from the same cellar (García, Aleixandre, Guti Rrez, & Horrillo, 2006; García, Fern Ndez, et al., 2006); identifying wines by geographical origin (Cynkar, Dambergs, Smith, & Cozzolino, 2010); and different wines coming from the same region (Aleixandre et al., 2008).

The most frequently used of the sensing unit in E‐nose systems reported is the metal oxide sensors (MOS; Kutsanedzie, Guo, & Chen, 2019). However, the MOS‐based E‐nose is bedeviled with many challenges, including being negatively affected by high temperature, excessive power demand, sulfur and weak acid toxicity, limited sensor coatings, humidity sensitivity, and low accuracy (Han, Huang, Aheto, Zhang, & Feng, 2020; Wilson & Baietto, 2009). Red wine is liquid in nature and so it is a challenge to quickly distinguish the volatile species while avoiding intrusions owing to changes in humidity using MOS.

Recently the E‐nose sensing unit has been implemented with colorimetric sensors as a solution to the afore‐mentioned constraints associated with the MOS‐based E‐nose. The colorimetric sensors utilize chemical dyes that are optically sensitive to food odors (Rakow & Suslick, 2000). Unique color change profiles are obtained when the colorimetric sensors react with VOCs through coordinated mechanism, acid and base interactions, dipolar, *π–π* molecular complexation, van der Waals interlink ages, and adsorption based on physical properties (Han et al., 2020). The E‐nose fabricated using colorimetric sensors array has several advantages compared to E‐nose based on MOS. Some of these advantages include (a) influence of humidity is eliminated; (b) simple and low cost; (c) convenient operations; and (d) various chromogenic dyes for every sensing material could be obtained. As a result, colorimetric sensor‐based E‐nose has therefore been widely used in the development of rapid analytical methods for ensuring food safety. For liquid foods, the feasibility of colorimetric sensor‐base E‐nose has indeed been evaluated for tracking the fermentation process of vinegar and grading of vinegar with specific specified ages (Chen, Liu, Zhao, Qin, & Lin, 2013; Chen et al., 2014). A groundbreaking artificial olfaction technology, centered on colorimetric sensors, was developed and utilized in the wine industry for the categorization of Chinese rice wine as per different defined ages (Ouyang, Zhao, Chen, & Lin, 2013). However, the application of colorimetric sensor‐based E‐nose for rapid quality evaluation of red wine has not been reported.

In case of sensory evaluation, signals of the receptors of olfactory and gustatory are collected and integrated to provide a final judgment of food quality. Use of E‐nose and E‐tongue integration is expected to provide more detailed knowledge about food consistency than information obtained individually from the two systems.

In the wine industry, analytical techniques premised on the integration of E‐nose and E‐tongue has been developed for deterioration assessment (Gil‐Sánchez et al., 2011), sensorial descriptors (Buratti, Ballabio, Benedetti, & Cosio, 2007), multimodal characterization (Rodríguez‐Méndez et al., 2004), and quantification of relevant compounds (Di Natale et al., 2000). Several reviews on this part could be found in previous studies (Loutfi, Coradeschi, Mani, Shankar, & Rayappan, 2015; Persaud, 2016; Rodríguez‐Méndez et al., 2016). Among the E‐tongues applied for the analysis of wine quality published till now, taste sensors based on potentiometry and voltammetry are the most commonly used. Compared with potentiometric sensors, the voltammetric E‐tongue has several special advantages, including high sensitivity, versatility, simplicity, and robustness and it has been extensively used in analytical chemistry (Tian, Deng, Ding, Yin, & Li, 2007).

The present study focused on the use of inexpensive E‐Nose fabricated with colorimetric sensors coupled with a commercial voltammetric E‐Tongue to classify red wines varying in geographical origins, brands, and grape varieties. Principal component analysis (PCA) was utilized for dimension reduction and decorrelation of the raw sensors datasets. Extreme learning machine (ELM) models were built with combination of the PCA scores of E‐nose and E‐tongue as the inputs.

## 2 MATERIALS AND METHODS

### 2.1 Red wine samples

Electronic nose and tongue tests were performed on a sample collection of eighty‐four red wines produced in China with three different geographical origins (Yantai, Langfang, and Xianyang), brands (Zhangyu, Great Wall, and Pengzhu), and grape varieties (Cabernet Sauvignon, Snake Dragon Ball, and Merlot). The red wines collected were bottled in 375 ml glass bottles. The detail information of the red wines used in this present study is presented in Table 1. All red wines were maintained in a refrigerator at 4°C with corks once the bottles were opened during experiments. Red wines from the same brand (Zhangyu) and grape variety (cabernet sauvignon) were measured to build model for identification of the geographical origins of the test wines; Red wines from the same geographical origin (Yantai) and grape variety (cabernet sauvignon) were used for build models for prediction of brands of the test wines; Red wines from the same geographical origin (Yantai) and brand (Zhangyu), but differ in grape varieties (Cabernet Sauvignon, Cabernet Girnischt, and Merlot) were measured and utilized to build models for identification of wines grape varieties.

**TABLE 1:** Red wines list with brands, origins, and grape varieties used for this study

Brands	Origins	Grape varieties	Bottles	Labels	Manufacturers
Zhangyu	Langfang, Hebei province	Cabernet Sauvignon	12	ZLC	Yantai Changyu Pioneer Wine Co., Ltd.
Zhangyu	Xianyang, Shanxi province	Cabernet Sauvignon	12	ZXC	
Zhangyu	Yantai, Shandong province	Cabernet Sauvignon	12	ZYC	
Zhangyu	Yantai, Shandong province	Miller	12	ZYM	
Zhangyu	Yantai, Shandong province	Cabernet Girnischt	12	ZYS	
Changcheng	Yantai, Shandong province	Cabernet Sauvignon	12	CYC	Cofco Great Wall Winery (Yantai) Co., Ltd.
Pengzhu	Yantai, Shandong province	Cabernet Sauvignon	12	PYC	Yantai Pengzhu Wine Co., Ltd.

### 2.2 E‐nose construction and data extraction

Colorimetric sensors in the E‐nose system developed were utilized for sensing of the VOCs released by red wine. Hence, fabrication of the chromogenic sensors array using specified chemical stains was the essential part for construction of the novel E‐nose system. Chemical dyes selected for the colorimetric sensors were premised on two fundamental criteria: (a) each chemical sensitive dye must have a center strongly interacted with analytes and (b) the interface center must be closely linked to a strong chromophore (Chen et al., 2016). The first criterion means that the interaction does not involve simple physical adsorption but other, greater chemical interactions. Chemo‐responsive colors are the colors that change in either reflected or absorbed light following changes in their chemical environment (Zou, Li, Shi, Huang, & Zhao, 2012). Porphyrins and their metal complexes are the most frequently used broad‐spectrum chemical dyes for VOCs sensing resulting from their open coordination sites for axial ligation, large spectral shifts upon ligand‐binding, and intense coloration (Huang, Xin, & Zhao, 2011).

In fabricating the colorimetric sensors array for the E‐nose system, twelve commercially available porphyrins and metalloporphyrins that are used frequently were selected, including:

1. 5,10,15,20‐tetraphenyl‐21h,23h‐porphine iron(iii)chloride;
2. 5,10,15,20‐Tetraphenyl‐21H,23H‐porphine;
3. 5,10,15,20‐Tetrakis(pentafluorophenyl)porphyrin iron(III) chloride;
4. 5,10,15,20‐Tetraphenylporphinatozinc;
5. 5,10,15,20‐Tetraphenyl‐21H,23H‐porphine manganese(III) chloride;
6. [5,10,15,20‐Tetraphenylporphinato(2‐)]copper;
7. 2,3,7,8,12,13,17,18‐octaethyl‐21H,23H‐porphine mn;
8. 5,10,15,20‐Tetraphenyl‐21H,23H‐porphine cobalt (II);
9. Zinc 2,3,9,10,16,17,23,24‐octakis(octyloxy)‐29H,31H‐phthalocyanine;
10. 5,10,15,20‐Tetra(4‐methoxyphenyl)porphyrin;
11. 5,10,15,20‐Tetrakis(4‐aminophenyl)porphyrin;
12. 5,10,15,20‐Tetrakis(4‐pyridyl)‐21H,23H‐porphyrin.

Additionally, three common pH indicators, namely methyl red, bromocresol green, bromocresol purple were added primarily to monitor the acidic ingredients. Nile red was also been added owing to its solvatochromic properties of changing optical characteristics upon sensing VOCs in addition to having exceptional stability to temperature and watervapour (Han et al., 2020). All dyes were purchased from Sigma‐Aldrich Chemical Co.

Subsequently, solutions of the selected chemical dyes with a concentration of 2 mg/ml were prepared. Chloroform was used as the solvent for preparation of the solutions of porphyrins and metalloporphyrins. More so, ethanol and acetone were used to dissolve the common pH pointers and Nile red dyes, respectively. The solvents used were of high grades, procured from from Sinopharm Chemical Reagent Co. Ltd. During the experiment, the mixtures of dyes and solvents were preprocessed for 30 min by ultra‐sonication at room temperature.

Finally, the sensor arrays were developed by marking the chemo‐responsive dyes on silica gel plates in reverse phase (Qingdao Puke Parting Materials Co. Ltd.) by means of microcapillary pipettes (0.1 µl). The colorimetric sensor arrays were kept in a nitrogen‐flushed glove bag after printing until measurements were required (Han et al., 2020).

**FIGURE 1:**
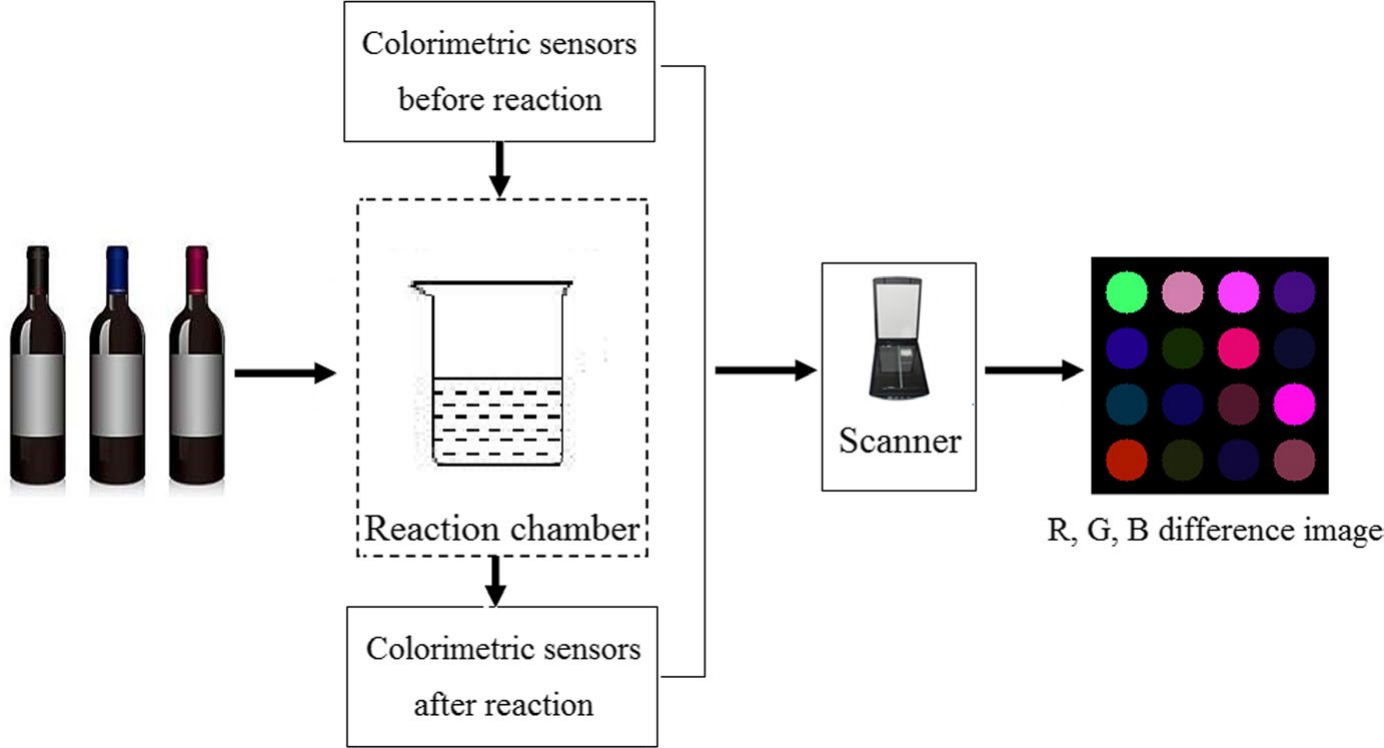
The flow chart of the electronic nose measurements for red wines. ELM, extreme learning machine

In this present study, the HP Scanjet 4,890 flatbed scanner (Hewlett Packard Inc.) was used as the color images acquisition instrument. Based on a pre‐experiment for the colorimetric measurements, 150 ml of one red wine sample was measured into a glass container (400 ml) and seal airtight. During colorimetric measurements, the surface area of one red wine sample was about 60 cm^2^, and the distance between the colorimetric sensors array and the liquid surface of the red wine sample was about 2.2 cm. The flow chart of the E‐nose measurements is presented in Figure 1. Measurements of E‐nose for red wine were performed at room temperature.

Images of the colorimetric sensors array were captured twice for each trial, before and 5 min after reaction with the VOCs released by red wines. A unique color change profile was obtained by subtracting the “after” image from the “before” image after the RGB (Red, Green, Blue) value change of each dyes was measured by averaging the center of each sensor spot (a total of about 450 pixels covering 12 pixel radiuses) to avoid factitious uniformity (Han et al., 2020). A 3N‐dimensional vector of the color shift profile used to evaluate by following chemometric approaches (where *N* = 16, number of sensors) was obtained (Han et al., 2020).

### 2.3 E‐tongue setup and operating steps

A commercially available E‐tongue (Isenso, Shanghai Ruifen International Trading Co., Ltd.) based on voltammetry coupled with a multifrequency large‐amplitude pulse scanner was employed for sensing the taste of the red wines. The E‐tongue adopts a standard three‐electrode system, namely working electrodes (platinum, gold, palladium, titanium, tungsten, and nickel), reference electrode (Ag/AgCl), and a platinum counter electrode. The applied potential is made up of three parts of 1, 10, and 100 Hz. The waveform of each segment of the large‐amplitude pulse waveform had the maximal value at 1.0 V and the minimal value at –1.0 V. A current was measured between the working electrode and the counter electrode when a voltage was applied over the working electrode and the reference electrode with the amplitude of each pulse was 0.2 V (Tian, Deng, & Chen, 2007).

**FIGURE 2:**
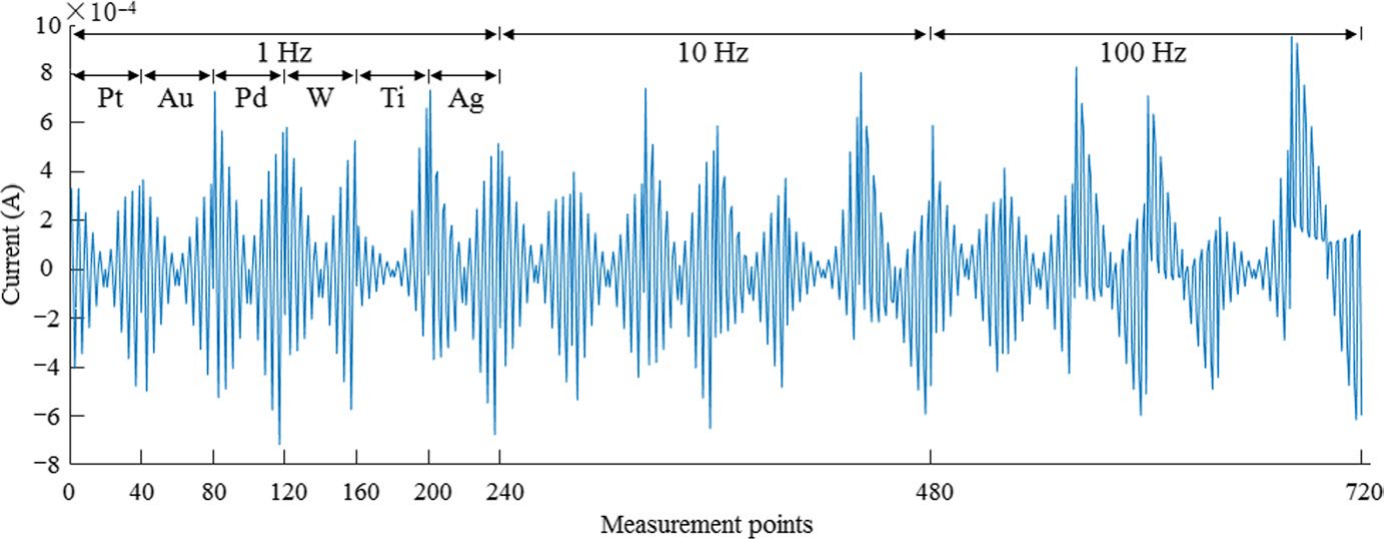
The characteristic values of electronic tongue electrodes for one red wine sample

In this work, 15 ml of one red wine sample was stirred and poured into the beaker (25 ml) for the E‐tongue measurements. Distilled water was used to clean the E‐tongue electrodes between each two wine sample measurement. The detection time of each wine sample was 26 s (1 Hz, 23 s; 10 Hz, 2.3 s; 100 Hz, 0.23 s). Four points within every loop were obtained as the characteristic values of one working electrode in relation to the concentration and dispersion coefficients of the charged and electro‐active compounds in the test solution as illustrated in Figure 2 for further data analysis (Tian, Deng, & Chen, 2007).

### 2.4 Data analysis approach and software

In this present study, decorrelation and dimensionality reduction of the characteristic variables obtained for E‐nose and tongue were performed by PCA. Then, mutually exclusive as well as the integration of PCA scores of E‐nose and tongue were used as inputs for the establishment of ELM models for identification of the red wines.

#### 2.4.1 Theory of the ELM

The ELM is a novel efficient learning algorithm based on single‐hidden layer feed a forward neural network which gives a good generalization performance at extremely fast learning speed. Compared with the traditional algorithms with gradient descent, dynamic adjustment for parameters is not necessary in ELM, which benefits from parameters of the input weight and the bias are generated randomly. As long as the neuron numbers in the hidden layer was set, the unique optimal solution for the pattern prediction could be obtained. Owing to its fast learning pace and good performance in generalization, ELMs have attracted the attentions of researchers in the field of food science and engineering (Geng, Zhao, Tao, & Han, 2017; Han, Huang, & Teye, 2019; Qiu & Wang, 2017).

Training of the ELM model could be divided into three steps (Yulei, 2015). Step 1: Randomly generates the input weight matrix and the bias matrix; Step 2: Select a transfer function, such as sigmoidal, sin, and hardlim, frequently used in the artificial neural network. Step 3: Calculate out the connection weight between the hidden layer and the output layer.

Afterward, the decision function for multi classification is,

(1) $$\left(x\right)=\underset{i\in \left\{1,2,\ldots ,m\right\}}{\arg\;\max\;T_{i}\left(x\right)},$$

where: *x* is the unknown sample for classification, *m* represents labels of the training samples, *T* is the output of the neural network.

Finally, performances of the ELM models built were evaluated by the recognition rate (%) calculated via correct predicted number over total number of measurements (Han et al., 2014).

#### 2.4.2 Software

Algorithms of PCA and ELM utilized in this present work were implemented in Matlab Version 7.14 (Mathworks) using Windows 7.

## 3 RESULTS AND DISCUSSION

### 3.1 General chemical analysis

Total acidity, volatile acidity, total sugar, alcohol content, and dry extract of the red wines used were analyzed following the Chinese standards (GB/T 15038‐2006). Results of the chemical analysis are collected in Table 2 to demonstrate the differences in basic chemical compositions of the red wine samples.

**TABLE 2:** Chemical parameters measured in red wines used

Chemical parameters	ZLC	ZXC	ZYC	ZYM	ZYS	CYC	PYC
Total acidity (g/L)	4.54 ± 0.02^a^	4.44 ± 0.02^b^	4.07 ± 0.02^c^	4.34 ± 0.01^d^	4.45 ± 0.03^b^	4.65 ± 0.02^e^	4.92 ± 0.02^f^
Volatile acidity (g/L)	0.53 ± 0.01^a^	0.54 ± 0.01^a^	0.50 ± 0.01^b^	0.43 ± 0.01^c^	0.49 ± 0.01^b^	0.44 ± 0.02^c^	0.36 ± 0.02^d^
Total sugar (g/L)	10.37 ± 0.06^a^	7.24 ± 0.07^b^	10.47 ± 0.04^a^	6.87 ± 0.06^c^	7.98 ± 0.07^d^	12.42 ± 0.01^e^	10.52 ± 0.1^a^
Alcohol content (%vol)	11.82 ± 0.02^a^	13.58 ± 0.01^b^	11.58 ± 0.02^c^	12.98 ± 0.02^d^	12.58 ± 0 0.03^e^	14.32 ± 0.19^f^	12.07 ± 0.04^g^
Dry extract (g/L)	15.9 ± 0^a^	23.2 ± 0.61^b^	16.9 ± 0^c^	15.9 ± 0.12^d^	16.2 ± 0^e^	17.0 ± 0^f^	11.8 ± 0.12^g^

Note

Results are expressed as *M* ± *SD*, *n* = 3. Values in the same line with different superscripts were significantly different (*p* < .05).

### 3.2 Decorrelation and dimensionality reduction of the E‐nose and tongue outputs

High‐dimensional and high‐collinearity of input variables could increase the complexity of a multivariate model and reduce its performance. In this study, PCA was executed firstly on the datasets of E‐nose and tongue, respectively, for decorrelation and dimensionality reduction of the original variables. PCA maps the original variables to a set of new features, defined by the principal components (PCs). The first PC is the direction of the largest variance of the original data matrix, the second PC explains the second‐largest variance and orthogonalized to the first PC, and the third PC explains the third‐largest variance and orthogonalized to the first and second PCs. By analogy, several PCs less than the original variables could be obtained. As a result, most of the variance is contained in the top several PCs, and the variance of the subsequent PCs is almost zero. For further modeling, the subsequent PCs could be ignored and only retain the top several PCs with most of the variance to substitute for the original variables. Principal components are linear and uncorrelated variables of the original dataset. Hence, decorrelation could be also obtained via PCA analysis.

**FIGURE 3:**
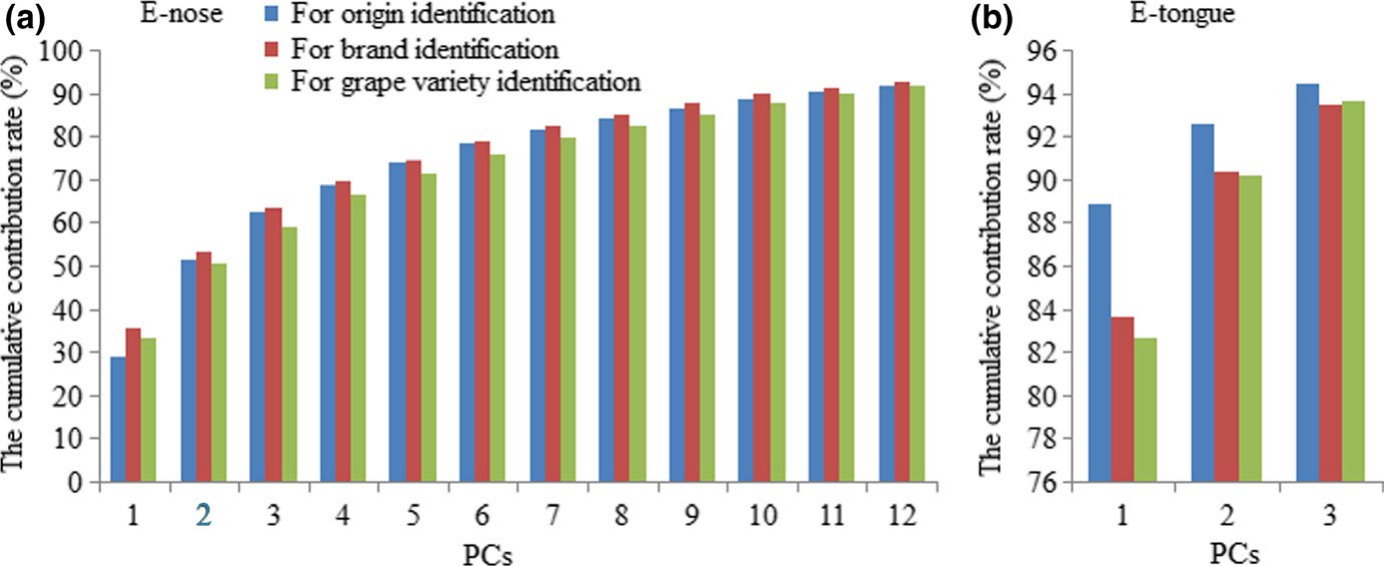
The cumulative contribution rates of top several PCs of the E‐nose (a) and tongue (b) data matrixes for red wines identification. E‐nose, electronic nose; E‐tongue, electronic tongue; PC, principal component

Figure 3 illustrates the total combined contribution rates of highest PCs of E‐nose and tongue datasets for red wines identification. It can be observed from the figure that the first 12 PCs and 3 PCs of the E‐nose and tongue datasets were sufficient to show their original variables, respectively, as more than 90% of the overall variance could be explained. Hence, the first 12 PCs and 3 PCs of the E‐nose and tongue were, respectively, obtained as the data points for establishing the ELM models.

The original variables of E‐nose and tongue collected exhibit obvious collinearity as presented in Figure 3. For E‐nose system, this phenomenon may be due to intense, broad responsiveness of the chemical dyes and broader sensitivity toward the complex VOCs of red wines. Either way, each dye in the sensor array may be sensitive to multiple volatile species at the same time and different dyes may also be sensitive to one of the species at the same time. Similarly, the metallic electrodes used in E‐tongue system also revealed weak selectivity and cross sensitivity to the soluble chemicals in liquid phase of the test wines, which resulted in a collinear output. It could be observed that E‐nose and E‐tongue are not like the traditional target detection analytical instruments (e.g., gas chromatography and gas chromatography–mass spectrometry), rather, they, respectively, have holistic response for the diverse volatiles or soluble taste components of foods.

### 3.3 E‐nose for red wines identification

In this section, algorithm of ELM was performed on E‐nose dataset to build prediction models for red wines. One third of each group's observations were stochastically chosen as the prediction set, and the remaining samples were considered the training set to predict geographical origins, brands, and grape varieties of red wines. During ELM modeling, the sigmoid function as shown in Equation (2) was used as the activation function of the hidden layers.

(2) $$S\left(x\right)=\frac{1}{1+e^{‐x}}.$$

**FIGURE 4:**
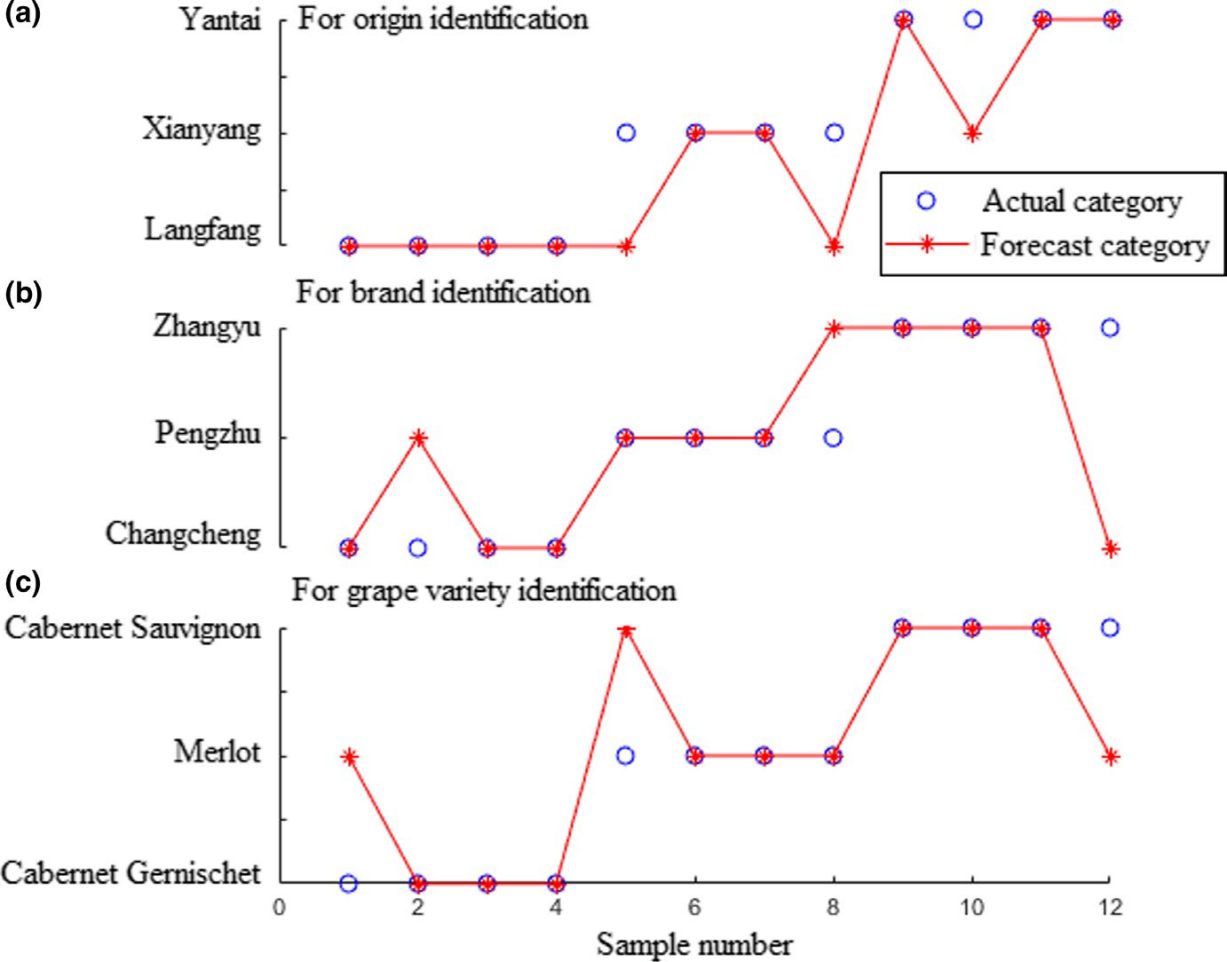
Results of the extreme learning machine models for identification of origins (a), brands (b), and grape varieties (c) of red wines using electronic nose

Based on the theory of ELM, the number of hidden neurons has a vital impact on its performance. In this present study, the number of hidden neurons was optimized by the maximum of the recognition rate in the prediction set. In order to avoid the scenario of over fitting for ELM models, two important principles were considered, (a) recognition rates were similar between in the training and prediction set; and (b) recognition rate in the training set is higher than that in the prediction set. Results showed that when the numbers of hidden neurons were 11, 10, and 17 (for geographical origins, brands, and grape varieties, respectively) the ELM models obtain the optimal prediction performance illustrated in Figure 4. As it can be observed, three samples were misclassified in each group. Recognition rate of 75% was achieved in the prediction set each for geographical origins, brands, and grape varieties of red wines.

**FIGURE 5:**
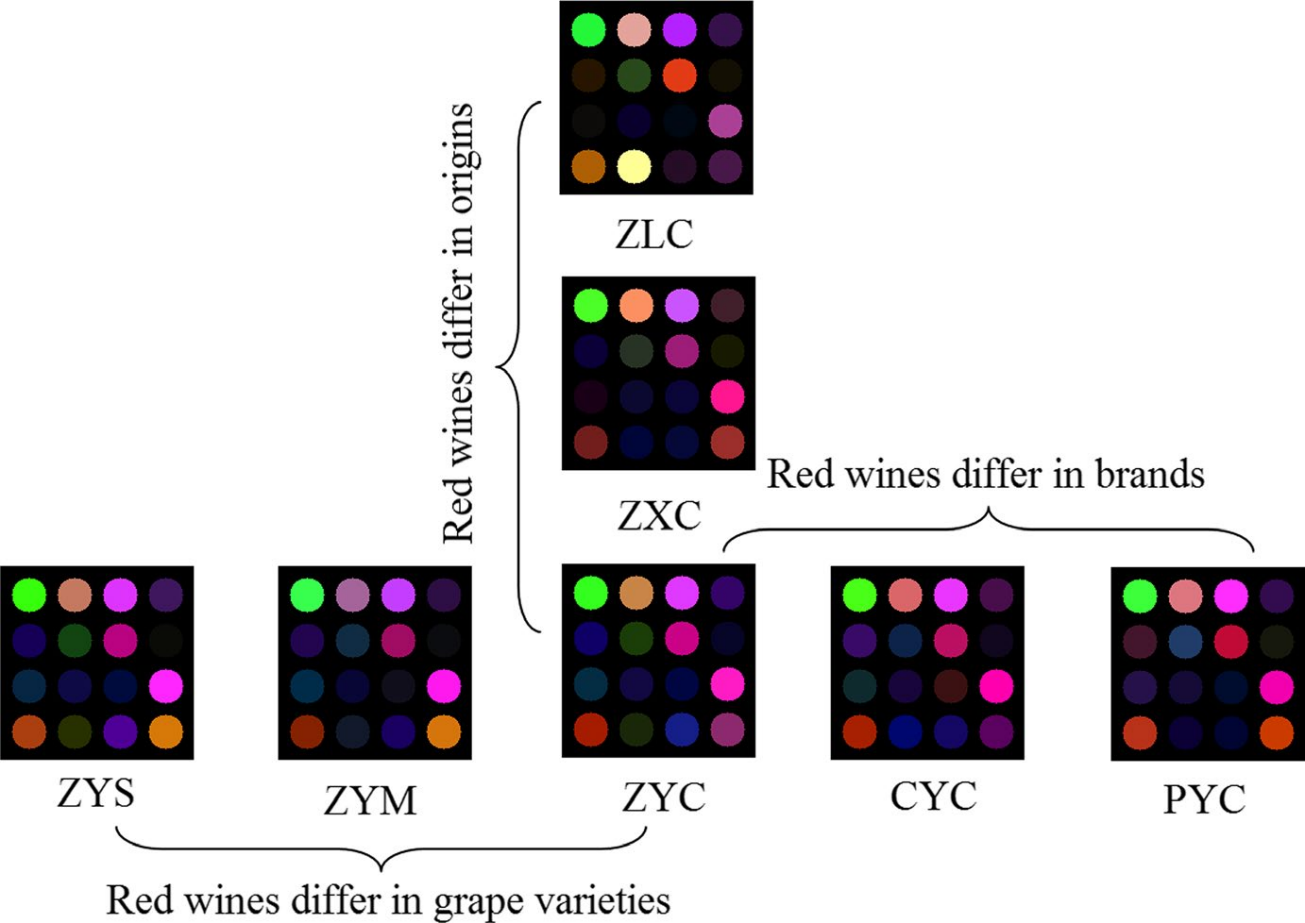
The RGB difference images of the electronic nose sensors for red wines identification

Figure 5 provides different RGB images of the E‐nose sensors for red wines showing variations in geographical origins, brands, and grape varieties. As it can be observed, sensor response generated a typical chemical fingerprint of the VOCs present in the red wines, which implied that the characteristic of the volatile compounds released by red wines` was closely linked to colorific changes of the selected sensitive dyes.

Volatile organic compounds of red wines originate mainly from the raw grape material, followed by production phases of alcoholic fermentation and wine aging. Previous researches have reported a total of over 1,000 volatile compounds in wines, of which alcohols, esters, aldehydes, and ketones feature strongly (Song et al., 2015). Many factors including the grape variety, climatice conditions, vineyard management practices, among others influence the aroma attribute of wine (Ziółkowska, Wąsowicz, & Jeleń, 2016). There are differences in species and concentrations of the VOCs released by red wines which differ in geographical origins, brands, and grape varieties, that is the basis of identification of these wines according to VOCs.

On the other hand, the porphyrins and their metal complexes used for the colorimetric sensors have accessible harmonization sites for asymmetric conjugation and strong coloration and can respond to organic molecules via acid–base interactions, bond formation, *π*–*π* molecular complexation, or van der Waals interaction and physical adsorption, then lead to their color transforms (Chen et al., 2013). The common pH indicators used also can change their optical properties with the change of volatile organic acid in VOCs released from red wines. In addition, the Nile red's non‐negative solvatochromic features makes it well positioned to assess the polarities of the VOCs through color changes (Han et al., 2020). These afore‐mentioned qualities can ensure that the behavior of the VOCs of interest is related to colorific change of chemical responsive dyes on the colorimetric sensor array. Therefore, the varying trends of the sensor array fingerprints keep in line with the wine VOCs varying trends (Han et al., 2020).

### 3.4 E‐tongue for red wines identification

**FIGURE 6:**
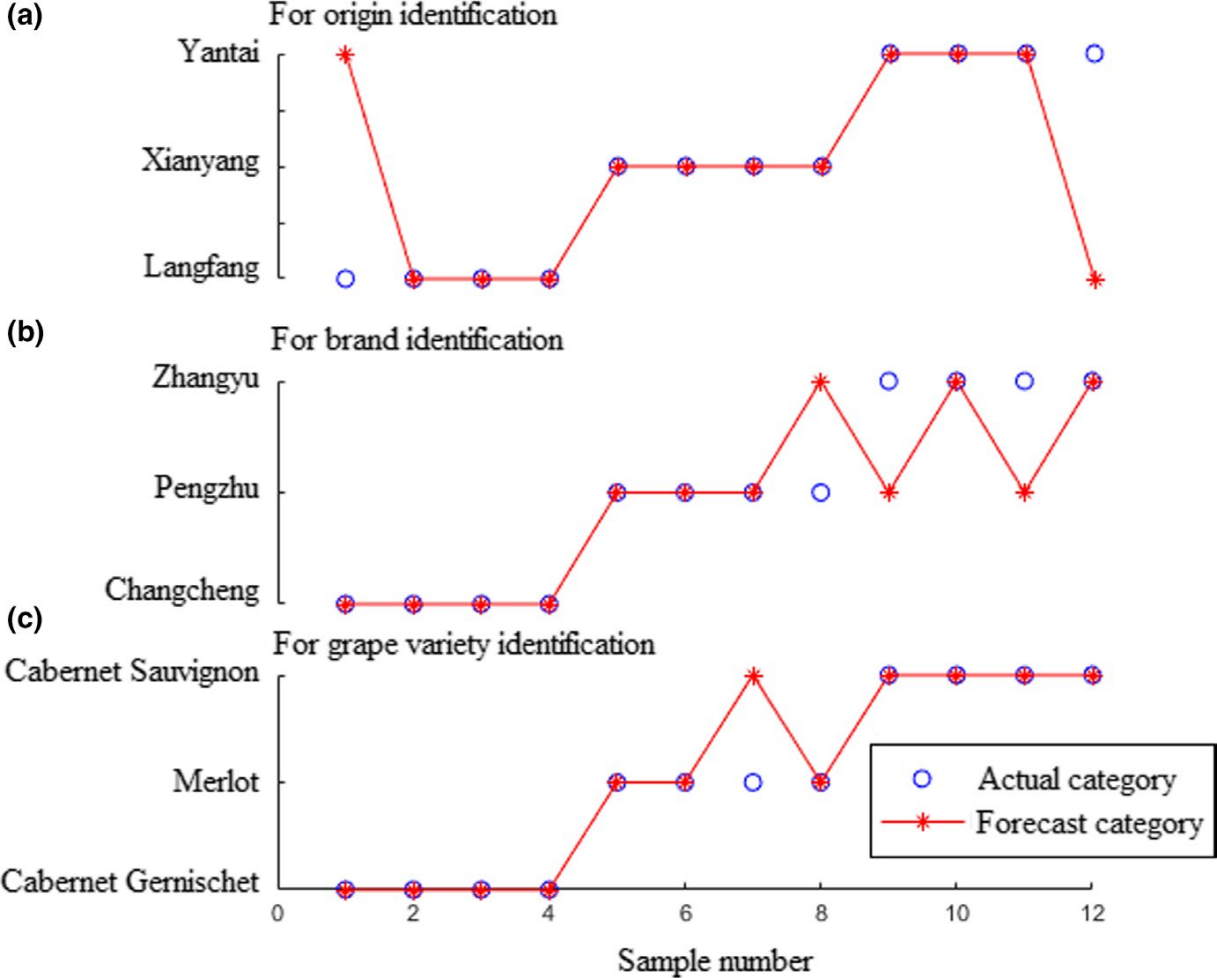
Results of the extreme learning machine models for identification of origins (a), brands (b), and grape varieties (c) of red wines using electronic tongue

In this work, no datasets produced by the E‐nose sensors were removed to keep useful signals as much as possible for red wines identification. Disadvantages of the high‐dimensional variables for modeling have been solved by PCA. A third of each group's samples were stochastically selected as the prediction set and the remaining samples as the training set. Sigmoid function was chosen as the hidden layers of ELM's activation function. Results show that when the numbers of hidden neurons were 10, 9, and 4 for prediction of origins, brands, and grape varieties of red wines, the ELM models obtain the optimal prediction performance showed in Figure 6. It could be observed from the figure that, two, three, and one samples were misclassified with the corresponding recognition rates were 83.33%, 75%, and 91.67% for prediction of origins, brands, and grape varieties of the wine samples, respectively.

The differences in grape varieties and origins constituted the main part of the differences in wine chemical components. Also the difference of production technology increased the change in wine chemical composition. The content of any one or several components cannot reflect the overall characteristics of wine. The broad multi‐sensitivity of the E‐tongue's metallic electrodes is used to evaluate the varying wine ingredients, which ensure that important "chemical images" can be extracted from the sensor array signals (Legin et al., 2003). Because each sensor offers a distinct pattern when inserted in each red wine solution, it can be assumed that the voltammograms could be used to differentiate and identify the selected samples using multivariate data analysis.

### 3.5 Fusion of E‐nose and tongue for red wines identification

In this section, signals produced by E‐nose and tongue were fused for red wines identification with the PCA scores as the inputs for ELM modeling. Results showed that all types of wines are correctly identified. These results indicated that this new methodology is able to carry out a significant classification of red wines according to their geographical origins, brands, and grape varieties. Table 3 shows the performances of the ELM models for red wines identification using E‐nose and tongue separately and their fusion.

**TABLE 3:** The recognition rates (%) of ELM models for identification of red wines differ in origins, brands, and grape varieties by E‐nose, E‐tongue, and fusion of them

	E‐nose		E‐tongue		E‐nose and tongue	
	Training set (%)	Prediction set (%)	Training set (%)	Prediction set (%)	Training set (%)	Prediction set (%)
Origins	79.17	75	87.5	83.33	100	100
Brands	83.33	75	79.17	75	100	100
Grape varieties	91.67	75	91.67	91.67	100	100
Totally	84.72	75	86.11	83.33	100	100

Abbreviations: ELM, extreme learning machine; E‐nose, electronic nose; E‐tongue, electronic tongue.

As tabulated in Table 3, the combination of E‐nose and tongue can effectively predict the tested wines according to their geographical origins, brands, and grape varieties. Furthermore, the performance of the ELM models based on the fusion technology was better than predicting these attributes using datasets of E‐nose and tongue separately. This is because the sensors of E‐nose and tongue obtain quality information of red wines from different aspects. Electrochemical information within the red wines is obtained by E‐tongue, while aroma information of red wines is obtained by the E‐nose.

## 4 CONCLUSIONS

A multi‐sensor fusion technology based on a novel low‐cost E‐nose and a voltammetric E‐tongue was developed to classify red wines that differ in geographical origins, brands, and grape varieties. The E‐nose was designed to transform the chemical information of wine aroma profiles into the quantitative color changes by the aid of colorimetric sensors. While, the commercial voltammetric E‐Tongue was employed to recognize the differences in taste of red wines. The fusion approach based on ELM models were built with combined PCA scores of E‐nose and tongue as the inputs. The optimum recognition rate of 100% for red wines was achieved in all instances. It can be concluded that the low‐cost E‐nose and voltammetric E‐tongue information fusion in conjunction with ELM was able to classify the wines tested, which has great guiding significance for monitoring red wine quality.

## CONFLICT OF INTEREST

The authors declare no conflict of interest.
